# Microwave Synthesized 2D WO_3_ Nanosheets for VOCs Gas Sensors

**DOI:** 10.3390/nano12183211

**Published:** 2022-09-15

**Authors:** He Liu, Lingyao Duan, Kedong Xia, Yang Chen, Yunling Li, Shaoxin Deng, Jiaqiang Xu, Zhenyu Hou

**Affiliations:** 1 School of Chemistry and Chemical Engineering, Henan Institute of Science and Technology, Xinxiang 453003, China; 2 Shanghai Yaolu Instrument & Equipment Co., Ltd., Shanghai 200444, China; 3 NEST Lab, Department of Physics, Department of Chemistry, College of Sciences, Shanghai University, Shanghai 200444, China

**Keywords:** microwave hydrothermal, nanosheets, sensing performance, oxygen vacancy

## Abstract

As an n-type semiconductor material, tungsten oxide (WO_3_) has good application prospects in the field of gas sensing. Herein, using oxalic acid (OA), citric acid (CA) and tartaric acid (TA) as auxiliary agents, three homogeneous tungsten oxide nanosheets were prepared by the rapid microwave-assisted hydrothermal method. The potential exhaled gases of various diseases were screened for the gas sensitivity test. Compared with WO_3_-OA and WO_3_-TA, WO_3_-CA exhibits significant sensitivity to formaldehyde, acetone and various alkanes. Photoluminescence (PL) chromatography and photoelectric properties show that its excellent gas sensitivity is due to its abundant oxygen vacancies and high surface charge migration rate, which can provide more preferential reaction sites with gas molecules. The experiment is of great significance for the sensor selection of the large disease exhaled gas sensor array.

## 1. Introduction

The detection and identification of organic pollutants are receiving large amounts of attention. Some volatile organic compounds (VOCs) are the target gases of pollution [1], and even the exhaled gas of cancer patients also contains some VOCs components [2], of which ketones, aldehydes and alkanes are the most common. VOCs have been used as biomarkers of some diseases or cancers, and they have been applied in the diagnosis and treatment of lung cancer, liver disease, breast cancer, gastric cancer, asthma, heart disease, diabetes and other diseases [3,4,5,6,7,8]. For example, acetone can diagnose diabetes when the exhaled gas concentration is higher than that of the standard value of healthy people [4]. Thirteen components such as nonane and undecane in exhaled gas have been identified as biomarkers of breast cancer [5]. Furfural, 1,2,3-trimethylbenzene and other gases have been found in the exhaled gas of gastric cancer patients [6]. Hua et al. [7] found that some volatile organic compounds in exhaled gas may screen for lung cancer, especially alkanes and aldehydes, including decane, undecane, n-heptaldehyde, etc. Modern analytical techniques such as gas chromatography-mass spectrometry (GC-MS), proton transfer reaction mass spectrometry (PTR-MS) and other methods can be used for gas analysis and determination [8]. However, the bionic electronic nose sensing technology based on nano-semiconductor materials has the advantage of overall determination, which can realize the overall identification of a variety of biomarkers in exhaled gas. It can avoid the failure to detect the content of a biomarker due to the low sensitivity of a single sensitive element, so it is more suitable to use the overall detection of exhaled gas to realize the rapid screening of diseases, and it has good promise in the rapid screening of diseases.

WO_3_ has been extensively studied for its good stability, suitable band gap and non-toxic properties [9]. Researchers have synthesized WO_3_ with different morphologies, including nanosheets [10,11,12,13,14], nanorods [15,16], nanotubes [17,18], nanowires [19] and hollow microspheres [20,21], among which the performance of two-dimensional (2D) structures is often superior to that of 1D and 3D structures. Two-dimensional (2D) nanosheets are stacked into mesoporous structures, which is conducive to the diffusion of gas molecules. Although there are numerous reports about the hydrothermal synthesis of WO_3_, the ordinary hydrothermal method has a long heating cycle, and it often takes several hours to synthesize WO_3_. The microwave-assisted hydrothermal synthesis method can improve the preparation process and make the particle size uniform. It is faster than the traditional method and has broad application prospects. Mehta et al. [22] provided a rapid and efficient microwave preparation method to obtain WO_3_ with layered structures for acetone sensing devices. Cavalcante et al. [23] heat-treated SrMoO_4_ powder with microwave water at 140 °C and observed PL at different time chamber temperatures, which were related to the strength and surface defects. Liquid phase microwave heating can improve the photocatalytic activity of WO_3_ [24,25]. Therefore, microwave-assisted synthesis also has the potential to improve the properties of functional materials [22]. Surface defects in oxide semiconductors can significantly accelerate surface reactions, facilitate charge separation, and adjust band structures to improve surface properties such as catalysis, adsorption, and gas sensing [26]. Typically, 2D nanosheet structures facilitate electron separation due to defects in oxygen vacancies on the surface [27]. For example, Ma et al. [28] proposed that increasing oxygen vacancies could increase oxygen absorption and reduce the activation energy of WO_3_. Tong et al. [29] prepared a high-performance gas sensor by using p-type copper ferrate CuCrO_2_ with enhanced oxygen vacancy defects.

In this paper, within 30 min, WO_3_ nanosheets were prepared by microwave-assisted hydrothermal synthesis with OA, CA and TA as carboxylic acid auxiliary agents. According to PL, the electrochemical impedance spectroscopy (EIS) and transient photocurrent phenomena, WO_3_-CA has higher sensitivity and response–recovery performance in gas sensing tests compared to WO_3_-TA and WO_3_-OA due to the role of surface oxygen vacancies and high surface charge migration rate. More than a dozen volatile organic compounds were tested to explore the gas sensitivity and application prospects of synthetic materials for exhaled gas biomarkers of serious diseases. Compared with WO_3_-OA and WO_3_-TA, WO_3_-CA nanosheets shows good gas sensitivity to formaldehyde, acetone and alkanes and other VOCs.

## 2. Materials and Methods

### 2.1. Materials

A reagent grade sodium tungstate dihydrate (Na_2_WO_4_·2H_2_O, 99.5%), citric acid (C_6_H_8_O_7_·H_2_O, 99.5%), oxalic acid (C_2_H_2_O_4_·2H_2_O, 99.5%), tartaric acid (C_4_H_6_O_6_, 95%, Shanghai Macklin Biochemical Co., Ltd., Shanghai, China), hydrochloric acid (HCl, 37%, Da Mao Chemical Reagent Factory, Tianjin, China) and ethanol (C_2_H_5_OH, 99.5%) were used directly without further purification. All reagents except those indicated are from Sinopharm Chemical Reagent Co., Ltd., (Shanghai, China).

### 2.2. Synthesis

The sodium tungstate dihydrate (Na_2_WO_4_·2H_2_O) and OA with a molar ratio of 2:1 were dissolved in 40 mL of distilled water. After stirring for 20 min at room temperature, 2.5 mL of hydrochloric acid was added, which was followed by further stirring for another 10 min. The solution was transferred to a 100 mL Teflon-lined reactor and heated by microwave at 200 °C for 30 min. Finally, the sample was washed with deionized water and ethanol and dried at 80 °C for 12 h. The sample was recorded as WO_3_-OA. Na_2_WO_4_·2H_2_O/CA and Na_2_WO_4_·2H_2_O/TA with the molar ratio of 1:1 were dissolved in 30 mL of distilled water, respectively. The other synthesis steps were the same as above, and these two samples were denoted as WO_3_-CA and WO_3_-TA, respectively.

### 2.3. Characterization

The crystal structure was investigated by X-ray diffraction (XRD, DX-2700B, Hao Yuan Instrument, Dandong, China). The morphological features of the obtained WO_3_ nanoarchitectures were investigated by field emission scanning electron microscope (FESEM, Regulus 8220, Hitachi, Tokyo, Japan) and field emission transmission electron microscope (FETEM, FEI Tecnai G2 f20, FEI company, Hillsboro, OR, USA). Nitrogen (N_2_) adsorption–desorption was conducted through Specific surface and pore structure analyzer (3H-2000PS2, BSD instrument, Beijing, China). Brunauer–Emmett–Teller (BET) schemes were used to calculate the specific surface area. The selected batches were degassed under vacuum at 150 °C for 6 h prior to BET analysis. The absorption spectra were analyzed with a double beam UV–vis spectrophotometer (UV, TU-1901, Beijing General Analytical Instrument, Beijing, China). UV–vis DRS spectra were recorded with BaSO_4_ as a reference chemical. The gas sensitivity characteristics of sensitive materials to target gas were tested by the CGS-8 intelligent gas sensitivity analysis system. X-ray photoelectron spectroscopy (XPS) measurements were conducted on a Thermo Fisher Scientific spectrometer (XPS, ESCALAB250Xi, Thermo Scientific K-Alpha, Waltham, MA, USA) employing Al K Alpha radiation. PL spectra were recorded with an Agilent Cary Eclipse fluorescence spectrophotometer (Agilent Technologies, Santa Clara, CA, USA).

### 2.4. Gas Sensing Measurement

Firstly, an appropriate amount of organic support (terpineol, ethyl cellulose) was added in 5 mg of WO_3_ powder to form a uniform slurry and then coated on the ceramic electrode. We heated it at 500 °C for 2 h to remove moisture and organic carrier and put it into an AS-20 aging system at 125 mA for 24 h. For the gas sensitivity test, a 500 mL gas chamber was used for static air distribution. When the resistance (R_a_) of the sensor material in air was basically stable, the target gas was introduced into the gas chamber with a micro sampler. When the baseline was stabilized again, the stability value was R_g_. The resistance response value is expressed as R_a_/R_g_.

## 3. Results and Discussion

### 3.1. Structure Analysis

**Figure 1:**
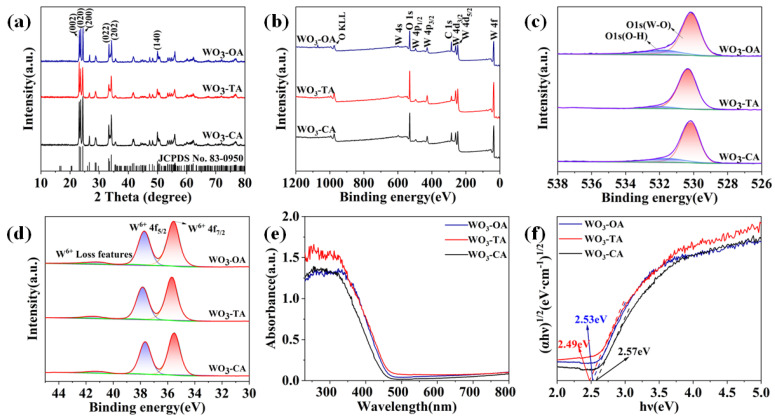
(**a**) XRD patterns, (**b**) The full-scale XPS spectra, (**c**) O1s, (**d**) W4f, (**e**) UV-vis absorption spectra of different samples, (**f**) Plots of the (ahν) ^1/2^ vs. photon energy (hν) for WO_3_ samples.

**Figure 2:**
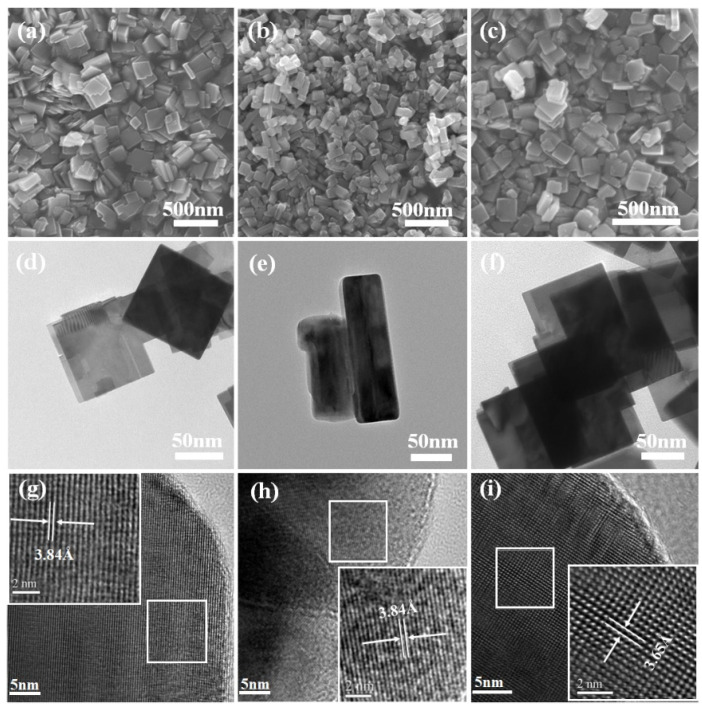
SEM, TEM and HRTEM images of (**a**,**d**,**g**) WO_3_-OA, (**b**,**e**,**h**) WO_3_-TA and (**c**,**f**,**i**) WO_3_-CA.

The XRD pattern exhibits the characteristic peak of monoclinic WO_3_ (JCPDS No.83-0950). Figure 1a shows that the addition of different auxiliary agents changed the relative intensity of the (002), (020), and (200) crystal planes, reflecting the change of sample morphology. The highest peak diffraction intensity in WO_3_-TA and WO_3_-OA was corresponding to the (002) and (200) crystal planes, respectively, which is consistent with the HRTEM results in Figure 2. The main exposed crystal planes of WO_3_ are crystal plane (002) and crystal plane (200), respectively. To further explore the relative content of the three main peaks, the respective peak areas of the (002), (020) and (200) planes were calculated by integrating the peak areas of XRD patterns (Figure S1 Supplementary Materials), and then, we calculated the percentage of the three crystal planes. The proportions of the (002) peak area of WO_3_-OA, WO_3_-TA and WO_3_-CA are 33.54%, 32.03% and 34.05%, respectively, and the proportions of (200) peak area are 34.46%, 35.18% and 33.86%, respectively. The results indicate that WO_3_-CA has a high (002) crystal surface content. The other two samples have the highest content of the crystal plane (200). XPS was used to analyze the valence states and chemical structures of elements. The full XPS spectrum of WO_3_ is illustrated in Figure 1b, and the S orbital and P orbital of the W atom suggest the characteristic features of W^6+^. The O 1s spectrum is fitted into two peaks (Figure 1c). The fitted peak at 530.2 eV is attributed to the W-O bond in WO_3_, and the peak at 531.8 eV is attributed to the hydroxide on its surface [30]. The W spectrum shows loss features at 41.06 eV, 41.42 eV and 41.07 eV, respectively (Figure 1d). The locations of WO_3_-OA, WO_3_-TA and WO_3_-CA are 37.7 eV and 35.5 eV, 37.8 eV and 35.6 eV, and 37.7 eV and 35.6 eV, respectively, both corresponding to orbital peaks of W^6+^ 4f_5/2_ and W^6+^ 4f_7/2_. All W 4f and O 1s regions are consistent with the reported XPS spectra of tungsten trioxide, indicating only W^6+^ is present in the synthesized WO_3_. Figure 1e and f show the UV-visible diffuse reflectance spectra and the optical band gap of three samples. The *E_g_* of OA, TA and CA is 2.53 eV, 2.49 eV and 2.57 eV at 400 nm, respectively. The difference between OA, TA and CA is 0.04 eV, which can be explained by the nano-size effect and impurity level transition caused by the crystal plane distortion and lattice defect [31].

### 3.2. Morphology Analysis

SEM images show that WO_3_-OA is a mostly irregular stacked sheet structure with a length of 150–300 nm and a thickness of about 25–30 nm. WO_3_-TA samples are about 80–200 nm in length and 50 nm in width, which are rectangular nanosheets with a thickness of 30–40 nm (Figure S2, Supplementary Materials). The morphology and size of WO_3_-CA are similar to WO_3_-OA, but the thickness is larger, about 40–45 nm. The crystal plane spacing of WO_3_-OA and WO_3_-TA is 0.384 nm, corresponding to the (002) crystal plane, while the (200) crystal plane spacing of WO_3_-CA is 0.365 nm. WO_3_-CA lattice stripes clearly show (200) crystal planes, which is consistent with XRD results. Otherwise, lamellar WO_3_ could not be obtained without an adjuvant. (Figure S2, Supplementary Materials). The three organic acids act as auxiliary agents and favor for the formation of the WO_3_ flake.

### 3.3. Gas Sensing Performance and Photoelectric Property

**Figure 3:**
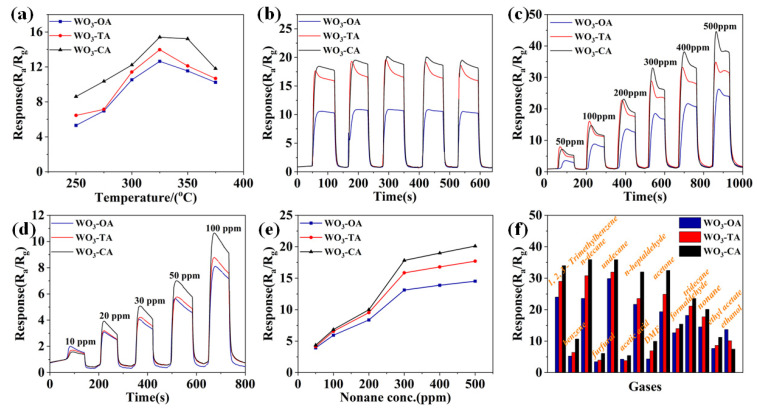
Gas sensitivity test at 35–50% relative humidity: (**a**) Working temperature and sensitivity; (**b**) Stability test at 300 ppm of acetone; (**c**) Decane response–recovery curve; (**d**) The response–recovery curve of undecane at lower concentrations; (**e**) The relationship between the sensitivity and the gas concentration in a nonane atmosphere; (**f**) Response values for 500 ppm VOCs gas at optimal operating temperature.

The formaldehyde response diagram with working temperature is shown in Figure 3a. The gas sensitivity increases gradually with the increase in temperature, and it maximizes at 325 °C. As the temperature continues to rise, the desorption rate is higher than the adsorption rate, resulting in fewer gas molecules adsorbed on the surface of the material, and the response speed slows down [32]. The optimal working temperature of other gases is consistent with that of formaldehyde, and the subsequent tests were all carried out at the 325 °C. The stability of three samples was tested in 300 ppm acetone, and all show the good repeatability (Figure 3b). Even at a higher decane concentration, there is still a faster response–recovery rate, among which WO_3_-CA has the highest sensitivity (Figure 3c). The higher the concentration of decane, the greater the difference in test sensitivity between the three samples. The response–recovery curve shows that the three sensors exhibit good responses at a lower undecane concentration (Figure 3d). WO_3_-CA still shows higher sensitivity than the other two. When the carrier density of the material reaches saturation, the response of nonane gradually slows down at high concentration (Figure 3e). The comparison diagram of the response values of different gas at 500 ppm (Figure 3f) shows the response of the three sensors to a variety of VOCs. It can be seen that the WO_3_-CA has high sensitivity to 1,2,3-trimethylbenzene, tridecane, undecane, decane, nonane, acetone, formaldehyde and n-heptaldehyde. In addition, the responses for benzene, furfural, acetic acid and N, N-dimethylformamide (DMF) were significantly lower than those gases, indicating the good selectivity of the sensor for these gases. It is necessary to add that isopropanol, methanol, propylene glycol and toluene have sensitivity values less than 3, as shown in Figure S3 (Supplementary Materials), indicating that the prepared sensor has better selectivity for these gases.

The sensitivity of gas sensors is not only related to the active site of the material but also to the adsorption and kinetic transport of the measured gas [33]. In the actual measurement process, adsorbed oxygen is activated to anion adsorbed oxygen. Only when the measured substance is close enough to the adsorbed anionic oxygen can electron transfer be carried out, showing the performance of gas sensitivity. However, different substances have different adsorption mechanisms due to their different polarity and steric hindrance. For non-polar molecules, since there is no electrostatic repulsion between them, they can better approach the oxygen adsorbed by anions, thus showing better gas-sensing properties. For polar molecules, there is a certain electrostatic repulsion between them and the anion-adsorbed oxygen, and it is more difficult to adsorb near the anion-adsorbed oxygen and reach the distance of electron transfer, thus affecting the sensitivity. Since WO_3_-CA has the most (002) crystalline surface content and the most anion-adsorbed oxygen [26], it has a greater electrostatic repulsive force to ethanol; therefore, for ethanol testing, WO_3_-CA shows a lower sensitivity.

**Figure 4:**
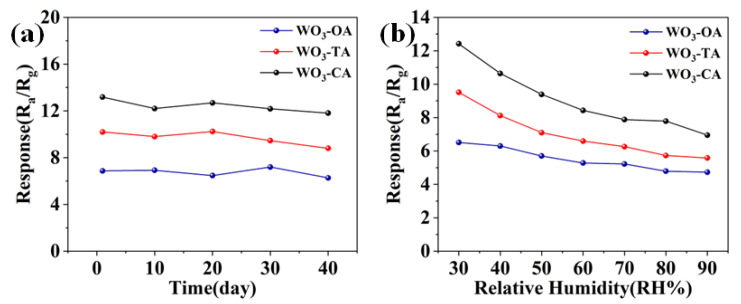
(**a**) Stability of the three WO_3_ in 100 ppm acetone and optimal operating temperature for 40 days. (**b**) Variation of acetone gas response at different relative humidity.

Long-term operating stability is an important indicator for testing sensor performance. The stability test was carried out with acetone at 598 K and 100 ppm gas concentration, and the samples were measured every ten days. The WO_3_-CA sensor still exhibits good response performance even after 40 days (Figure 4a), and it approaches 90% of the initial response value, indicating the good long-term stability of the sensor [34]. Furthermore, sensors are often in contact with humid environments in practical operating conditions, so it is also important to explore the effect of humidity on sensors. The sensitivity of the sensor was performed in acetone at 598 K,100 ppm, with different humidity environments (Figure 4b). With increasing relative humidity, water molecules may react with chemisorbed oxygen or adsorb on the metal oxide surface, and this competing adsorption [35] of acetone and water molecules at relatively high operating temperatures limits the availability of active sites for the adsorption of gas molecules, leading to a decrease in sensitivity [36,37].

**Figure 5:**
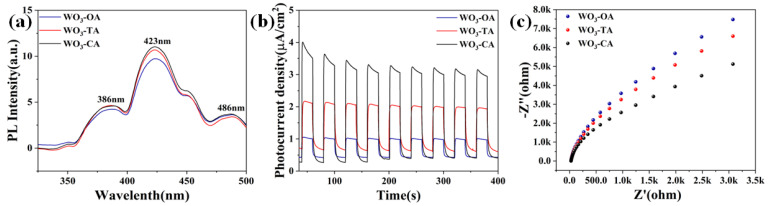
(**a**) The PL spectra of the different WO_3_ samples. (**b**,**c**) Transient photocurrent responses and EIS Nyquist plots.

The PL spectra of WO_3_ samples at an excitation wavelength of 321 nm exhibit the main emission band at 400–470 nm and the maximum peak at 423 nm (Figure 5a). The PL spectra in the blue and violet light region (423 nm) can be interpreted as crystal structure defects [38]. The emission of light in the visible region (486 nm) is attributed to the oxygen vacancy defect, and its intensity is positively correlated with the defect density [39], among which WO_3_-CA has the highest defect density. This enhanced defect feature leads to an increase in the number of reaction sites for gas adsorption. Since the oxygen vacancy defect is an active center for oxygen adsorption, more oxygen vacancy defects can adsorb more oxygen molecules to form a thicker electron depletion layer, leading to the increase in R_a_. At the same time, the redox reaction between the adsorbed oxygen and the measured gas is strengthened, leading to the reduction in R_g_. Therefore, the highest response of the WO_3_-CA gas sensing element could be caused by the maximum oxygen vacancy concentration [40].

In order to further investigate the reasons for the higher sensitivity of WO_3_-CA, photocurrent and EIS were used to detect the separation efficiency and the movement of charge carriers on the surface of the material. The photocurrent response shows rapid response during the switching cycle of simulated sunlight illumination (Figure 5b). Compared to WO_3_-TA and WO_3_-OA, WO_3_-CA shows higher photocurrent response changes. The EIS Nyquist plot exhibits that the impedance arc radius of WO_3_-CA is smaller than that of WO_3_-TA and WO_3_-OA, indicating a higher charge transfer efficiency (Figure 5c). The reason for this phenomenon may be due to the synergistic effect of exposed (200) crystal surfaces, oxygen vacancies and higher specific surface areas (WO_3_-OA:12.56 m^2^/g, WO_3_-TA:12.05 m^2^/g, WO_3_-CA:13.28 m^2^/g, Figure S4, Supplementary Materials). These results indicate that the higher surface electron activity means that surface electrons of WO_3_-CA materials can easily bind to free oxygen in air, thus improving gas sensitivity [27]. The specific surface area of WO_3_-CA is higher than that of WO_3_-TA and WO_3_-OA, which can provide more active sites and increase the contact area between the material and gas molecules, thus improving the gas sensitivity. Although the specific surface area of WO_3_-OA is larger than that of WO_3_-TA, this increased specific surface area is not sufficient to produce the high electrical response for which their synergistic results with tailored crystalline surfaces seem to be effective [41]. Therefore, the relatively small difference in the specific surface area between the three materials is explained by the synergistic effect between the three materials and the active crystal surface.

The crystal surface exposure of semiconductor metal oxides will affect their performance as gas sensors [42,43], The shape control order of the surface energy of WO_3_ nanocrystals with monoclinic structure is (002) (1.56 J m^−2^) > (020) (1.54 J m^−2^) > (200) (1.43 J m^−2^), indicating that (002) is the most active in the surface-mediated reaction [44]. In this work, the main exposed surface of the WO_3_-CA nanosheet is the (200) crystal surface, but its (002) crystal surface has a high content, which is conducive to oxygen atom adsorption in the air. There are a large number of W atoms on the (020) crystal surface, indicating that there are a large number of hanging bonds, which is conducive to sensor performance. The (200) plane consists of a mixture of O and W atoms, with the number of W atoms being smaller than that of the (020) plane [42]. Facet (200) has the advantage of easy chemisorption of oxygen and high reactivity of the suspended bond of W atoms [45]. The synergistic effect brought by the different crystal surface content, exposure surface, specific surface area, and oxygen vacancy concentration differences of the three WO_3_ types is the key to the excellent gas sensitivity of WO_3_-CA. The gas sensitivity mechanism is explained as follows.

C_n_H_2n+2_ (gas) + O^−^→C_n_H_2n+1_• + O^2−^ + H^+^; (1)

C_n_H_2n+1_• + O^−^→C_n_H_2n_ (gas)+ O^2−^ + H^+^; (2)

O^2−^ + 2H^+^→H_2_O (gas). (3)

Due to the chemisorption of gas molecules and the reaction with oxygen molecules, a depletion layer of oxygen anions is formed. The gas adsorption [46] on the surface of the material reacts with the adsorbed oxygen on the surface, liberating electrons to return. The above gas-sensitive mechanism is shown in Equations (1)–(3); taking alkane as an example, when the sensor material is exposed to a specific concentration of alkane atmosphere, the alkane molecules adsorb on the surface of the material. At a high temperature, the C-H bond at the end group of the alkane molecule breaks, forming a methylene group. The sensor surface captures hydrogen, forming a double bond between the alkene and the connected C atoms [47]. The adsorbed alkyl group can proceed to another mechanism, including the formation of surface oxide intermediates, which can be further oxidized to aldehydes and carboxylate and eventually to carbon oxides.

## 4. Conclusions

In this paper, WO_3_ nanosheets with different properties were prepared by the microwave hydrothermal method with three carboxylic acids as structural guides. The gas sensitivity of WO_3_ nanosheets was studied, and more than a dozen potential VOCs in exhaled gases from serious diseases were tested. The WO_3_-CA gas sensor is suitable for 1,2,3-trimethylbenzene, tridecane, undecane, decane, nonane, acetone, formaldehyde and n-heptaldehyde, with higher sensitivity, response recovery performance and stability, while it has certain selectivity with ethyl acetate, dimethylamine, methanol, isopropanol, toluene, furfural, acetic acid, DMF, benzene and ethanol. XRD, PL, EIS and transient photocurrent test results show that WO_3_-CA has more surface oxygen vacancy defects, higher surface charge mobility, and a possibly synergistic effect between the crystal surface and specific surface area and oxygen vacancy defects, which makes WO_3_-CA significantly better than WO_3_-OA and WO_3_-TA. This study provides a simple and rapid method for the synthesis of WO_3_ nanosheets, and the prepared sensor has an important reference value for the selection of alkane biomarker sensors in the exhaled gas sensor array.

## Data Availability

Not applicable.

## Supplementary Materials

The following supporting information can be downloaded at: https://www.mdpi.com/article/10.3390/nano12183211/s1, Figure S1: Peak area and full-width at the half of the maximum (FEHM) after XRD integration; (a) WO_3_-OA, (b) WO_3_-TA, (c) WO_3_-CA; Figure S2: SEM images of three samples without adjuvants, and sample thickness diagram (a,d) WO_3_-OA, (b,e) WO_3_-TA, (c,f) WO_3_-CA; Figure S3: Schematic diagram of three sensor selectivity with tridecane as control; Figure S4: N_2_ adsorption/desorption isotherms for (a) WO_3_-OA, (b) WO_3_-TA, (c) WO_3_-CA.
