# Supplementary material for: Microwave Synthesized 2D WO3 Nanosheets for VOCs Gas Sensors
Source: Nanomaterials (Basel). 2022 Sep 15;12(18):3211. doi: 10.3390/nano12183211 (PMC9506399; doi:10.3390/nano12183211)
Supplement: Supplementary file 1 [file nanomaterials-12-03211-s001.zip › nanomaterials-1896416-supplementary.pdf]

nanomaterials

Supplementary Materials

Microwave Synthesized 2D WO_3 Nanosheets for VOCs Gas Sensors

He Liu [1], Lingyao Duan [1], Kedong Xia [1], Yang Chen [2,3], Yunling Li [1], Shaoxin Deng [1], Jiaqiang Xu [3] and Zhenyu Hou [1,*]

[1] School of Chemistry and Chemical Engineering, Henan Institute of Science and Technology, Xinxiang 453003, China; heliu8043@163.com (H.L.); ddlingyao@163.com (L.D.); kdongxia@163.com (K.X.); liyl271@126.com (Y.L.); dengshaoxin123@126.com (S.D.)
[2] Shanghai Yaolu Instrument & Equipment Co., Ltd., Shanghai 200444, China; shucy@shu.edu.cn
[3] NEST Lab, Department of Physics, Department of Chemistry, College of Sciences, Shanghai University, Shanghai 200444, China; xujiaqiang@shu.edu.cn
* Correspondence: houzhy0373@163.com

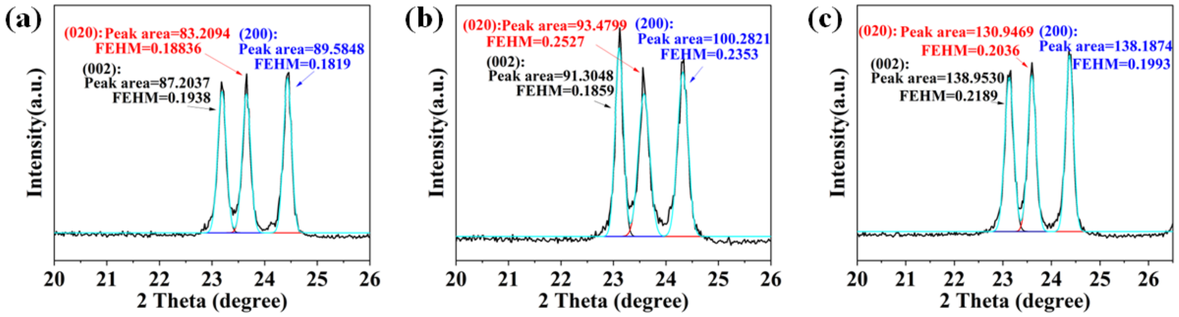

Figure S1. Peak area and Full-width at the half of the maximum (FEHM) after XRD integration; (**a**) WO_3-OA, (**b**) WO_3-TA, (**c**) WO_3-CA.

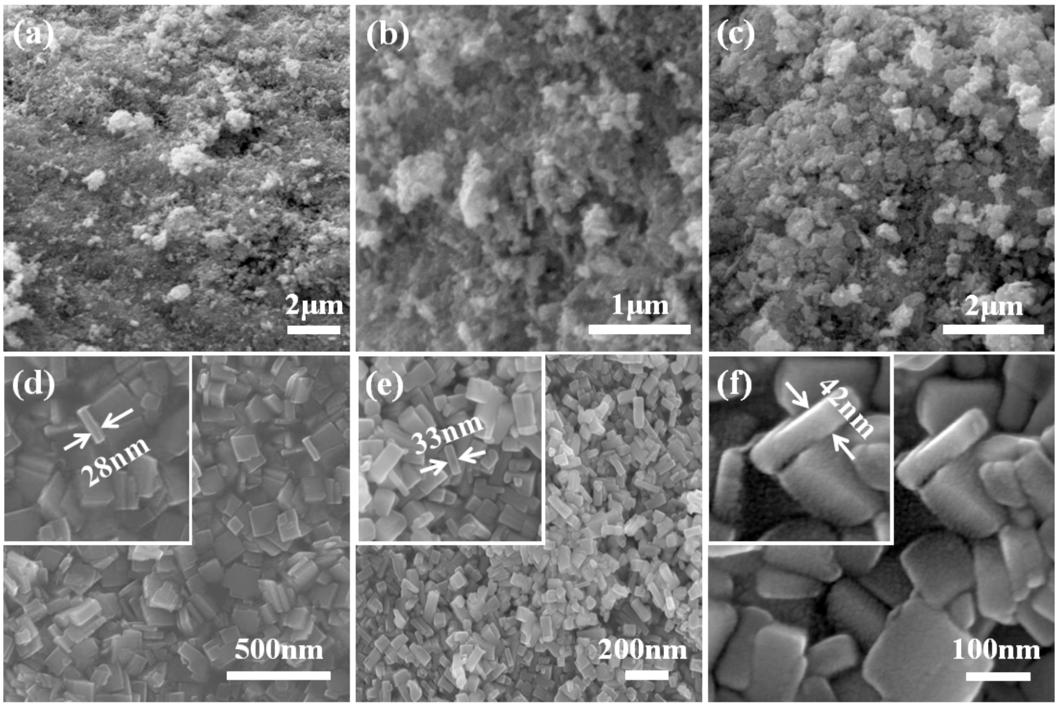

Figure S2. SEM images of three samples without adjuvants, and sample thickness diagram; (**a**,**d**) WO_3-OA, (**b**,**e**) WO_3-TA, (**c**,**f**) WO_3-CA.

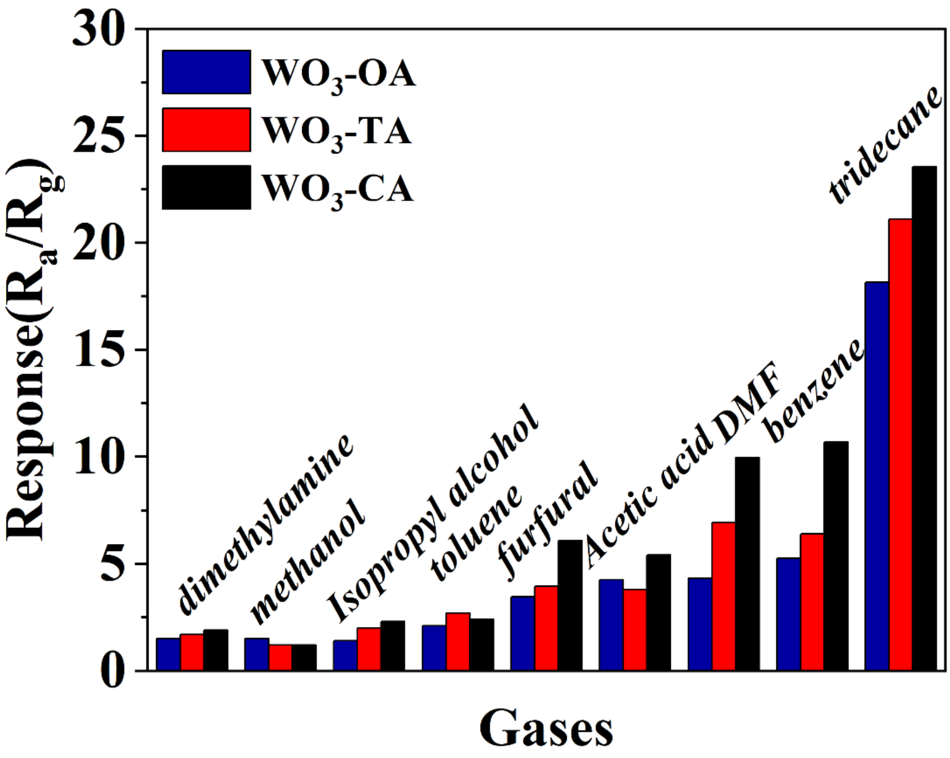

Figure S3. Schematic diagram of three sensor selectivity with tridecane as control.

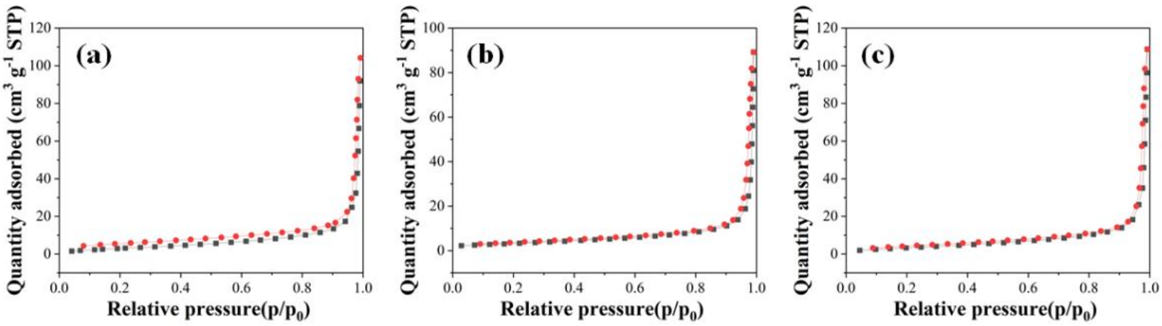

Figure S4. N_2 adsorption/desorption isotherms for (**a**) WO_3-OA, (**b**) WO_3-TA, (**c**) WO_3-CA.
